# The Sequence [RRKLPVGRS] Is a Nuclear Localization Signal for Importin 8 Binding (NLS8): A Chemical Biology and Bioinformatics Study

**DOI:** 10.3390/ijms26062814

**Published:** 2025-03-20

**Authors:** Athanasios A. Panagiotopoulos, Konstantina Kalyvianaki, Aikaterini Angelidaki, Dimitris Dellis, Christos A. Panagiotidis, Marilena Kampa, Elias Castanas

**Affiliations:** 1 Laboratory of Experimental Endocrinology, School of Medicine, University of Crete, Voutes Campus, 71013 Heraklion, Greece; medp2011839@med.uoc.gr (K.K.); kate.agg2003@gmail.com (A.A.); kampam@uoc.gr (M.K.); castanas@uoc.gr (E.C.); 2 National Infrastructures for Research and Technology, 11523 Athens, Greece; ntell@grnet.gr; 3 Laboratory of Pharmacology, School of Pharmacy, Aristotle University of Thessaloniki, 54124 Thessaloniki, Greece; pchristo@pharm.auth.gr

**Keywords:** importin 8, nuclear localization signal (NLS), karyopherins, RanGDP

## Abstract

Karyopherins, carrier proteins that recognize particular cargo protein patterns known as nuclear localization signals (NLSs), mediate the nuclear translocation of big proteins. In order to better understand the process of nuclear transport of proteins and create the groundwork for the development of innovative treatments that specifically target importins, it is imperative to clarify the intricate interactions between nuclear transporters and their cargo proteins. Until recently, very few NLSs have been documented. In the current work, an in silico method was used to identify NLSs for importin 8. It was determined that the sequence RRKLPVGRS serves as a recognition motif for importin 8 binding a karyopherin that is involved in the nuclear transportation of several important proteins like AGOs, SMADs, RPL23A, and TFE3. The sequence was validated in vitro in the breast cancer cell line T47D. This work subscribes to the effort to clarify the intricate relationships between nuclear transporters and their cargo proteins, in order to better understand the mechanism of nuclear transport of proteins and lay the groundwork for the development of novel therapeutics that target particular importins and have an immediate translational impact.

## 1. Introduction

Accurate cellular compartmentalization is essential to life in eukaryotic cells and a key component of their physiology is the shuttling of nucleo-cytoplasmic proteins [1,2,3]. Large proteins must be imported into the nucleus through aided cytoplasmic-nuclear exchange, which is mediated by specialized protein carriers, while small proteins and peptides may translocate through the nuclear pore by passive diffusion [1]. These transporters, known as karyopherins, are a family of at least 20 different proteins that belong to three different classes: importins, involved in cytoplasmic-nuclear trafficking; exportins, which are in charge of translocating nucleo-cytoplasmic proteins through the nuclear pore; and adaptor proteins, frequently required for the formation of the importin-cargo protein complex [1,4].

The small Ras-related GTPase Ran, which regulates importin-cargo complex construction and disassembly, is essential to importin-mediated nuclear transport [2]. The GTP- versus GDP-bound forms of Ran in the cytoplasm and nucleus dictate the way of nuclear-cytoplasmic transport [2,5]. Importin-cargo complexes are bound by Ran-GDP, which controls their cytoplasmic-nuclear transport [2,5]. Once inside the nucleus, the exchange of GDP with GTP controls the release of the transferred protein [2,5,6], which therefore regulates large protein transport to the nucleus and importin shuttle-tailing [2,7].

The Nuclear Localization Signal (NLS) is the recognition motif found in cargo proteins that allows them to attach to importins [8,9]. Until recently, only a few NLS motifs for importin α (IMPOα) and the M9 NLS (recognized by importin β2, also known as transportin) have been identified, despite significant scientific efforts [10,11,12,13,14,15,16]. A great deal of work went into identifying NLS sequences in a growing number of proteins, creating online tools to predict these motifs, and eventually predicting the nuclear translocation of proteins via the RanGDP–Importin α–Importin β (IMPOβ) system [14,17,18,19,20], which led to the development of particular medications [21,22,23,24]. Recently, the sequence EKRKI(E/R)(K/L/R/S/T) has been advanced as a recognition motif for binding with importin 7 through the use of a bio-informatics approach, based on bibliographic data, simulation, and experimental in vitro validation [25], while the sequences LPPRS(G/P)P and KP(K/Y)LV [26] were identified as recognition motifs for importins 4 and 5. Here, using a similar methodology, we report the sequence RRKLPVGRS as the recognition motif for importin 8. The importance of our research is in the identification of importin 8 as a key transporter that is involved in the nuclear import of certain proteins. Importin 8 differs from other importins in its cargo selectivity and biological functions [27]. In the present study, we present initial evidence that it mediates the nuclear import of functionally heterogeneous and clinically important proteins such as Argonaute proteins, SMADs, and zinc finger proteins [27]. The results of this study enhance the knowledge of nuclear transport mechanisms and may be relevant to therapeutic approaches targeting importin 8-mediated processes. Identification of RRKLPVGRS as an importin 8 recognition signal is a novel contribution, adding to the list of known NLS sequences and contributing to new knowledge on the molecular importin 8-mediated nuclear transport process.

## 2. Results

### 2.1. In Silico Characterization of Importin 8 NLSs

For the detection of importin 8 NLSs, we used the proteins AGO1 (Protein argonaute-1) [28,29,30,31,32,33], AGO2 (Protein argonaute-2) [34,35,36,37,38,39], AGO3 (Protein argonaute-3) [40,41,42], AGO4 (Protein argonaute-4) [43,44,45,46], RPL23A (Large ribosomal subunit protein uL23) [47,48], SMAD1 (Mothers against decapentaplegic homolog 1) [49,50,51], SMAD3 (Mothers against decapentaplegic homolog 3) [52,53,54], SRP19 (Signal recognition particle 19 kDa protein) [55,56,57], TFE3 (Transcription factor E3) [58,59,60,61], WT1 (Wilms tumor protein) [62,63,64], ZFP2 (Zinc finger protein ZFP2) [65], ZNF264 (Zinc finger protein 264) [66], and ZNF774 (Zinc finger protein 774) [67], previously reported to interact with importin 8 (Table 1).

The structures of the proteins were retrieved from the AlphaFold database (https://alphafold.ebi.ac.uk/, accessed on 2 May 2024) [68]. A comparison of the recently reported AlphaFold structures used here for binding with importin 8 with their previously reported crystal structures (Supplemental Table S1) [68] confirms the correct conformation of the 3D prediction we have used here.

**Figure 1:**
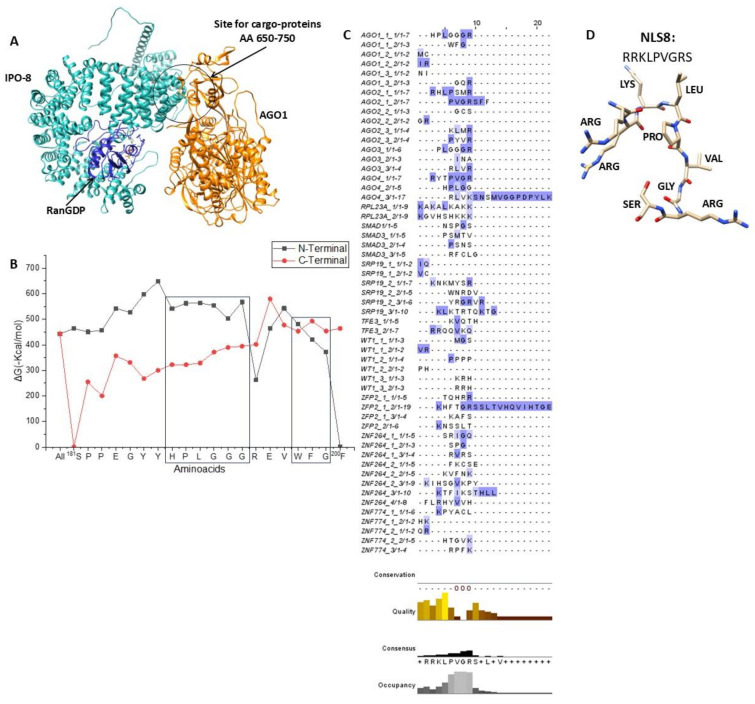
In silico identification of the importin 8-NLS motif. (**A**) Protein argonaute-1 (AGO1) (orange) docking on the blue-colored RanGDP-IPO8 heteroprotein complex (cyan). For additional information, see results. Following structure optimizations in the GalaxyWEB server [75,76,77], protein interactions were computed using the HEX 8.0.8 tool [78,79]. The UCSF Chimera program was used to create the image [80]. (**B**) Adjustment of the relationship between the AGO1-extracted peptide sequence and importin 8 (displayed in Table 1), calculated using HEX 8.0.8 [78,79], and presented as ΔG (Kcal/mol) values. The ΔG values of N-terminally shortened sequences are displayed in black curves, whereas the ΔG values of C-terminally truncated sequences are displayed in red curves. The NLS sequence prediction uses the retained peptide sequences, which are displayed in the black box. For more information, see the text. (**C**) Using the web tool Jalview, the minimum amino acid sequences (shown in (**B**) and Table 1) were aligned [81]. At the bottom is the consensus sequence that was retrieved. (**D**) A three-dimensional depiction of the minimal consensus sequence identified as an importin 8-NLS recognition motif.

As shown in Figure 1A, importin 8 interacted with the cargo proteins at amino acids 650–750, whereas it bound to the small GTPase Ran at amino acids 50–150. After importin binding to the previously listed proteins (found using the HEX 8.0.8 tool), the interacting amino acids were extracted, and are shown in Table 1.

Figure 1A provides a specific illustration of importin 8 binding to AGO1, and Supplemental Figure S1 displays importin 8 interaction with all aforementioned proteins. We found the minimal sequence that each protein uses to interact with importin 8 (Figure 1B and Supplemental Figures S2–S14) by recursively removing one amino acid at a time from the N- or C-terminal of the identified importin interacting peptide (see Section 4 and Ref. [25] for details).

The alignment of all identified motifs (Figure 1C) revealed the common sequence RRKLPVGRS as the NLS for importin 8 (Figure 1C), the deletion of which significantly reduces the protein–importin 8 binding affinity (Figure 1B). Figure 1D displays its three-dimensional configuration.

It is interesting to note that molecular docking study according to the above methodology (Supplemental Figure S15) showed that valine and glycine at positions 6 and 7, respectively, do not directly interfere with the binding of the cargo protein; however, their deletion results in a weaker binding to Importin 8. Nevertheless, as determined by in silico mutagenesis and alanine replacement, it appears that the presence of Leucine at position 4 interacts with and stabilizes the conformation of the NLS-related amino acids (particularly lysine and proline, at positions 3–5 of the NLS sequence).

**Table 1:** Proteins interacting with Importin 8. The protein short names, their AlphaFold codes, the corresponding references, the in silico predicted amino acid sequences interacting with importin 8, and the related Gibbs free energy changes (ΔG) of the interaction, are reported.

Cargo Protein	AlphaFold Code	References	Interacting Amino Acids	ΔG (Kcal/mol)
AGO1	AF-Q9UL18-F1	[69]	^181^SPPEGYYHPLGGGREVWFGF^200^	−442.91
			^228^VIEFMCEVLDIRN^240^	−298.03
			^336^YLPLEVCNIVAGQRC^350^	−590.03
AGO2	AF-Q9UKV8-F1	[69,70]	^166^MRHLPSMRYTPVGRSFFT^183^	−1106.31
			^185^SEGCSNPLGGGREVWF^200^	−470.15
			^380^SKLMRSASFNTDPYVRE^396^	−974.05
AGO3	AF-Q9H9G7-F1	[69]	^186^PEGYDHPLGGGREVW^200^	−302.12
			^359^DNQTSTMIKAT^369^	−446.97
			^373^APDRQEEISRLVRSA^387^	−592.07
AGO4	AF-Q9HCK5-F1	[69]	^162^MRYTPVGRSF^171^	−855.04
			^175^PEGYYHPLGGG^185^	−350.66
			^362^APDRQEEISRLVKSNSMVGGPDPYLKE^388^	−628.82
RPL23A	AF-P62750-F1	[71]	^10^PAPPKAEAKAKALKAKKAVLKGVHSHKKKK^39^	−2127.57
SMAD1	AF-Q15797-F1	[72]	^187^SPNSSYPNSPGSSS^199^	−609.18
SMAD3	AF-P84022-F1	[72]	^251^SQPSMTVDGFTDPSNSERFCLGL^273^	−352.31
SRP19	AF-P09132-F1	[71]	^45^TATEIQDVCSA^55^	−226.35
			^63^EKNKMYSREWNRDVQYRGRVRV^84^	−1307.71
			^111^MIPKLKTRTQKTGGAD^126^	−1125.31
TFE3	AF-P19532-F1	[73]	^173^PREVLKVQTHLENPTRYHLQQARRQQVKQYLSTTLGPKLASQ^214^	−1319.95
WT1	AF-P19544-F1	[74]	^1^MGSDVRDLN^9^	−380.46
			^58^PAPPPPPPPPPHSFIK^73^	−661.45
			^365^SRSDQLKRHQRRHT^378^	−1123.92
ZFP2	AF-Q6ZN57-F1	[74]	^322^GVKPFECNECGKAFSKNSSLTQHRRIHTGEKPYECMVCGKHFTGRSSLTVHQVIHTGEKPYECNECGKAFSQ^393^	−1439.80
			^418^AFIKNSSLTV^427^	−655.69
ZNF264	AF-O43296-F1	[74]	^169^SRIGQEQVSPGDRVRSH^185^	−714.25
			^201^NNFKCSECGKVFNKKHLLAGHEKIHSGVKPYEC^233^	−1203.69
			^236^CGKTFIKSTHLLQHH^250^	−853.09
			^327^RPGFLRHYVVHS^338^	−922.59
ZNF774	AF-Q6NX45-F1	[74]	^261^KPYACLECHKS^271^	−779.05
			^280^THQRTHTGVKPY^291^	−865.15
			^373^RPFKCENC^380^	−724.34

### 2.2. In Vitro Validation of RRKLPVGRS Sequence as the Importin 8 NLS

**Figure 2:**
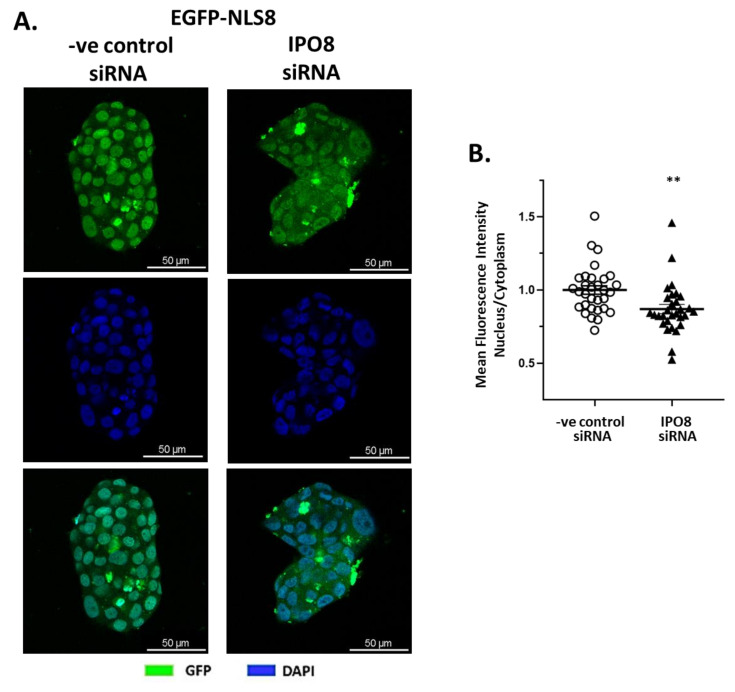
(**A**) Representative confocal pictures of T47D cells transfected with plasmid expressing the EGFP-NLS8 fusion (EGFP-NLS8), which is recognized by importin 8, in the presence of either a specific siRNA for importin 8 (IPO8) or a negative control siRNA. Nuclei were stained with DAPI (blue). Magnification 400×. (**B**) Mean fluorescence intensity in the nucleus and cytoplasm was quantified (see Section 4 for details) in at least 30 images with approximately 40 cells, per condition, and is given as the Nucleus/Cytoplasm fluorescence ratio. ** denotes statistical significance *p* < 0.01.

The in silico results for the NLS8 sequence were validated in vitro. For this, we constructed a plasmid that expressed the enhanced green fluorescent protein (EGFP) and had the NLS8 sequence attached to the protein’s C-terminus. We transfected T47D breast cancer cells which express importin 8 at high levels (MDA-MB-231 breast cancer cells and DU-145 prostate cancer cells had lower expression; Supplemental Figure S16), and observed EGFP cytoplasmic to nuclear presence by confocal microscopy (Figure 2). Confocal images clearly show a specific high EGFP fluorescence in the nucleus with the EGFP-NLS8 that is significantly reduced when importin 8 is knocked down with specific siRNAs (Supplemental Figure S17) (Figure 2A for representative confocal images and Figure 2B for the quantitation of nuclear and cytoplasmic staining).

## 3. Discussion

Nuclear import is a very selective process, based on particular import signals [8,10]. With a few notable exceptions, nuclear import signals are usually short sequences of amino acids that are located in the relevant protein’s DNA or RNA binding regions [82,83,84,85]. However, not much progress has been made in identifying other importin recognition signals, other than from importin α [10,11] and the M9 (transportin) NLS [12,13,14]. Here, we present the sequence RRKLPVGRS as a recognition site for importin 8, using the same in silico methodology followed by in vitro validation that we have previously applied for the identification of the NLS sequences for importin 4 ((L)PPRS(G/P)P), 5 (KP(K/Y)LV), and 7 (EKRKI(E/R)(K/L/R/S/T)) [25,26], with the latter also being verified by another group [86]. Here, we propose the sequence RRKLPVGRS as the protein recognition motif (NLS) for protein interactions with importin 8 (IPO8).

The identified importin 8 NLS mainly consists of polar amino acids like arginine, lysine, and serine; non-polar hydrophobic amino acids such as leucine; and aliphatic non-polar amino acids like valine. Additionally, it includes neutral amino acids like proline and glycine. As shown, this amino acid distribution suggests that the IPO8-NLS binding site recognizes polar sequences partially charged. It is worth noting that the NLS8 sequence shows binding selectivity to importin 8, because in corresponding in silico experiments, it was shown that its binding energy reached ΔG = −806.7 Kcal/mol, in contrast to importins α, β, 4, 5, 7, 9, 11, and 13, which showed no stable binding (ΔG > −250 Κcal/mol).

A structural analysis of NLSs allows us to understand the differences on the surface of importins. The presence of different positively charged amino acids at various positions may affect the interaction with the transported cargo, as well as the resistance or preference towards specific molecules or complexes. Indeed, NLS8 (RRKLPVGRS) contains a series of positively charged amino acids, such as arginine (R) and lysine (K), as well as a proline (P) residue, which appears to be characteristic of importin 8. This arrangement may affect the interaction with RNA–protein complexes, as reported in our study. NLS7 (EKRKI(E/R)(K/L/R/S/T)) contains a sequence that is more flexible due to multiple choices for the amino acid position at the end (E/R, K/L/R/S/T), which provides greater variety in the composition of the NLS. NLS4 (LPPRS(G/P)P) and NLS5 (KP(K/Y)LV) have fewer interactive characteristics than NLS8, with fewer positively charged amino acids and a more restricted sequence. The NLSα (KKKRK) is characteristic of many importins, with the presence of the positively charged KKKRK, which is often recognized by the transport system. The arrangement of positive amino acids (such as lysine and arginine) in NLSs can influence electrostatic interactions with the corresponding recognition protein systems. NLS8, as reported here, contains a denser cluster of positive charges, which may be important for the recognition of charges associated with small RNAs and protein complexes. In contrast, NLSα, which is used by many importins, has a more common electrostatic characteristic arrangement that may favor general charge recognition.

The proposed sequence of RRKLPVGRS as the protein recognition motif (NLS) for protein interactions with importin 8 (IPO8) was validated in vitro by using an EGFP protein construct containing in its C-terminal the proposed recognition sequence. Knocking down IPO8 with a specific siRNA, EGPF-NLS for importin 8 fluorescence in the nucleus was decreased, verifying the specificity of the proposed NLS sequence for IPO8.

We consider that this discovery might be of importance, as this karyopherin is implicated in major processes. IPO8, being a member of the importins family, plays an important role in transporting proteins into the cell nucleus [87,88]. More specifically, importin 8 functions as a receptor that recognizes and binds nuclear import sequence (NLS)-containing proteins, facilitating their passage through the nuclear pore and entry into the nucleus [88]. Additionally, IPO8 has unique characteristics that distinguishes it from other importins [88]. Among these are its involvement in the regulation of transcription and RNA processing, as well as the transport of specific regulatory proteins and RNA macromolecules [88]. For example, IPO8 has been found to be involved in the transport of complexes of small RNA molecules (miRNAs)—proteins involved in the regulation of gene expression [89]. While RNA molecules have negatively charged phosphate groups, the positively charged NLS of IPO8 likely facilitates the binding of IPO8 to other components of the RNA–protein complex, such as the associated regulatory proteins. This probably allows IPO8 to transport these complexes, rather than binding RNA directly.

Importin 8 mediates the nuclear import of several important proteins. Argonaute proteins 1–4 (AGO1–AGO4) [69] play a crucial role in gene expression regulation through RNA-mediated gene silencing and regulation by microRNA and small interfering RNA [69]. Argonaute proteins bind and target small RNAs, forming RNA–protein complexes known as RISCs, which recognize and bind target mRNA with complementary sequences [90]. After binding, Argonaute proteins can repress gene expression through degrading the target mRNA, suppressing translation, or inducing epigenetic modifications [91]. Argonaute proteins are essential for gene regulation, cell differentiation, growth, and defense against viruses [92]. RPL23A is a ribosomal component essential for protein biosynthesis, with mutations leading to disorders like anemia and cancer [93]. SMAD1 and SMAD3 mediate TGF-β (Transforming Growth Factor-beta) [94] signaling, influencing cell growth, differentiation, and apoptosis [95]. Mutations in SMAD3 are linked to Loeys–Dietz Syndrome, an inherited connective tissue disorder [27]. SRP19 is involved in the transport of newly synthesized proteins to the endoplasmic reticulum, with disturbances causing protein aggregation and organ dysfunction [57]. TFE3 regulates genes involved in autophagy and cell growth, with mutations associated with kidney cancer and Alveolar Soft Part Sarcoma (ASPS) [58]. WT1 is crucial for kidney and genital development, with mutations causing cancers like Wilms tumors and genital dysgenesis [96,97]. ZFP2 [98] and ZNF264 [99] are zinc finger proteins regulating gene expression; their dysfunctions can cause developmental abnormalities and cancer. ZNF774, another zinc finger protein, is linked to cell growth and neurological disorders when its function is disturbed [67].

Our method, which is comparable to the one previously described for the discovery of recognition signals for importins 4, 5 [26], and 7 [25], was verified by contrasting the anticipated cargo protein structures with those obtained by an independent approach (AlphaFold [68]) (see Supplemental Table S1).

It is well known that importins’ binding to the small GTPase Ran is what causes importin complexes to form containing cargos such proteins, RNAs, or RNPs intended for nuclear import [2,5,6,100,101]. The direction of importins’ movement—toward or out of the nucleus—is determined by the interaction between the GDP- and GTP-bound forms of Ran, bound to importins [2,5,6,100,101].

IPO8 [89,102] has been reported to interact with Ran, which is indispensable for its action. Also, we have simulated Ran-GDP interaction with IPO8 (Figure 1A), in view of the 3D prediction of the structure of the importin, bound to Ran-GDP. As expected for protein-protein interactions, the ΔG value was very high (ΔG = −2625.2 Kcal/mol), suggesting a rather stable interaction. Ran-GDP interacts with importins with similar binding affinities, as evidenced by comparisons of its interactions with importin β [25], importin 7 [25], importin 4 [26], importin 5 [26], and here, importin 8. However, more experimental research is required to validate the stability of the proposed complex.

Although our research is strong evidence that RRKLPVGRS is a novel nuclear localization signal for importin 8, we recognize some limitations in our method. Computer docking programs like HEX 8.0.8 are useful for providing information about protein–protein interactions; however, they are limited in their accuracy. Different docking programs can provide variation in the prediction of binding affinity. Despite this, the in vitro confirmation of our predicted NLS validates the integrity of our results and highlights the importance of using complementary techniques to study nuclear import mechanisms.

## 4. Materials and Methods

### 4.1. Bioinformatics Methods for Identification of the Ιmportin 8 NLS Sequence

Our group previously released an extensive description of the bio-informatics approaches developed to identify NLS motifs on cargo proteins [25]. In summary, the steps mentioned below were carried out.

#### 4.1.1. Selection and Generation of PDB Files

Protein sequences were imported into the Swiss Model Biospace (https://swissmodel.expasy.org/interactive, accessed on 2 May 2024) after being obtained in FASTA format from the NCBI protein database (https://www.ncbi.nlm.nih.gov/protein/, accessed on 2 May 2024) [103]. Protein databank (https://www.rcsb.org/, accessed on 2 May 2024) codes for the proteins were obtained [104]. The predicted model or models with 100% homology were the only ones retained. Small compounds that co-crystallized with the receptor in the single receptor crystal model were manually extracted from the PDB files using a text editor. The best model from the Swiss Model Biospace (https://swissmodel.expasy.org/interactive, accessed on 4 May 2024) was retained for proteins whose crystallized structures were unavailable [103]. This decision was made based on the sequence coverage homology (at least 70%) with a protein that has already been published. Structural templates were retrieved from the Protein Data Bank (PDB) without excluding NMR-derived structures, provided they met the criteria of sufficient sequence coverage (≥70%) and structural resolution appropriate for docking analyses.

Protein files (in PDB format) were refined in a fully flexible conformation and submitted to the Galaxy Refine server (https://galaxy.seoklab.org/, accessed on 6 May 2024; Routine REFINE) [75,76,77]. Using molecular dynamics simulations as a guide, REFINE reconstructs and optimizes the structure of side chains, secondary structural components, and loops [105].

The recently released AlphaFold database was compared with the 3D conformation of proteins [68]. To further evaluate the correctness of the predicted cargo protein structures, we compared the completed models derived using SwissModel and GalaxyRefine to the corresponding structures in the AlphaFold database. This extra validation was an independent verification of the overall fold and the accessibilities of critical surface regions that contribute to importin 8 binding. This cross-validation exercise enhanced our level of confidence in the structural compatibility of the identified NLS motifs and importin 8 binding interface. Whenever possible, Chimera software, version 1.14 (https://www.cgl.ucsf.edu/chimera/, accessed on 8 May 2024), was used to compare the flexible retained model with known crystals and AlphaFold published structures [80]. The three-dimensional structural differences were computed using Root Mean Square Differences (RMSDs) in Å for both individual areas and the entire molecule.

#### 4.1.2. Flexible Refined Solutions of Proteins Models

The Galaxy server’s LigDock and GalaxyHeteromer routines were used to perform Ran-GDP complex and importin 8 complex with Ran-GDP. The REFINE routine at the Galaxy server was used to refine the retained change in Gibbs free energy (ΔG) values. LigDock predicts the 3D structure for protein-ligand complexes, while GalaxyHeteromer uses template-based and ab initio docking to make 3D structure predictions for protein–protein complexes [106,107,108]. Both for importin and cargo proteins, the interaction (binding) interface was found, and the matching amino acids were obtained from the returned data. Using the GalaxyWeb server and routine TBM, the interacting amino acid structures in 3D for Importin 8 were modeled.

#### 4.1.3. Protein–Protein Binding Simulations

The interacting cargo protein sequence was also modeled in GalaxyWeb (routine TBM), and the Hex 8.0.8 program (https://hex.loria.fr/, accessed on 10 May 2024), in PDB format, was used to execute the (rigid) binding of the sequence [78,79]. The value of ΔG, or the change in Gibbs free energy, was obtained and documented. Following the removal of one amino acid from either the N- or C-terminus of each peptide (and reconstructing the remaining peptide in GalaxyWeb), the binding of the retained cargo sequences was repeated.

Following the final step of the process, ΔG graphs were generated for each docking simulation. Amino acids were retained as part of the proposed NLS if their removal led to a sharp increase in ΔG (>50 Kcal/mol), indicating their critical contribution to the stability of the importin 8-cargo complex (see Section 2 and Supplemental Material for specific examples).

#### 4.1.4. Peptide Sequence Alignment

Using the online resource Jalview (https://www.jalview.org, accessed on 26 May 2024), we finally aligned all of the preserved peptide sequences and obtained the consensus sequence for the binding of importin 8 [81].

### 4.2. In Vitro Methods for Validation of Ιmportin 8 NLS Sequence

#### 4.2.1. Cell Culture and Materials

The cell line T47D (purchased from DSMZ, Braunschweig, Germany) was cultured in RPMI-1640 (Gibco™, Thermo Fisher Scientific, Waltham, MA, USA) supplemented with 10% Fetal Bovine Serum and 1% penicillin-streptomycin (Qualified, Gibco™, Thermo Fischer Scientific), at 37 °C and 5% CO_2_. The selection of the T47D cell line was made as it has a high level of IPO8 (Importin 8) gene expression, as determined by real-time qPCR and The Human Protein Atlas Database (https://www.proteinatlas.org/, accessed on 17 October 2024). All media were purchased from Fisher Scientific (part of Thermo Fisher Scientific, Hampton, NH, USA), and all chemicals from Sigma (St. Louis, MO, USA), unless otherwise stated.

#### 4.2.2. Preparation of GFP-NLS8 Plasmid

The putative importin 8 recognition sequence, i.e., RRKLPVGRS, was fused to the carboxy-terminus of EGFP (EGFP-NLS8) using a process similar to that described in our previous publications [26,109]. Specifically, two oligonucleotides encoding the sequence of the putative NLS8 were synthesized and annealed in vitro, as previously described. The sequences of the two oligonucleotides were as follows: NLS8-corF: 5′-tcgaAGAAAGCTGCCTGTGGGCAGAAGCG-3′ and rNLS8: 5′-gatcCGCTTCTGCCCACAGGCA-GCTTTCT-3′, and they were designed in such a way as to generate single-stranded overhangs following their annealing that would allow for directional cloning in vectors digested with the *Xho*I and *Bam*HI restriction endonucleases. Note that these restriction enzyme site-compatible overhang sequences are in small letters. The annealed oligonucleotides carrying *Xho*I and *Bam*HI overhangs were ligated with plasmid pEGFP-C1 (Clontech) that had been digested with *Xho*I/*Bam*HI to generate plasmid pEGFP-C1-NLS8. The sequence of pEGFP-C1-NLS8, which expresses the putative NLS8 oligopeptide fused to the carboxy-terminus of EGFP, was verified by sequence analysis (CeMIA SA).

#### 4.2.3. Cell Transfection for GFP-NLS8 and IPO8 Silencing

Cells were seeded and incubated for 24 h at 37 °C, at an initial of 35 × 10^5^ cells/well in a 6-well plate, or 35 × 10^3^ cells/chamber in an 8-chamber slide. The specific plasmid pEGFP-NLS8 that expresses the RRKLPVGRS sequence (NLS8) was co-transfected using Attractene Transfection Reagent (QIAGEN, Hilden, Germany), with specific siRNAs (2 μg siRNA, 0.10 μg plasmid and 0.60 μL Attractene Transfection Reagent/10^4^ cells) for IPO8 (ID: 17813, Ambion, Thermo Fisher Scientific, Waltham, MA, USA) or negative control siRNAs (ID: 149,158 Silencer Select Negative Control #1 siRNA 4390843, Ambion, Thermo Fisher Scientific, Waltham, MA, USA). After 24 h, fresh medium was added, and, after another 24 h, cells were collected and analyzed with real time qPCR (plates) or fixed with 4% paraformaldehyde in PBS for 10 min (chamber slides).

#### 4.2.4. RNA Isolation and Real-Time PCR

In order to evaluate the transfection efficiency of siRNA, the total IPO8 gene expression was determined through real-time qPCR. Total cell RNA was isolated with the RNA isolation Kit (Nucleospin, Macherey-Nagel, Düren, Germany), cDNA, synthesized using the PrimeScript™ RT Kit (TaKaRa Bio Inc., Kusatsu, Shiga, Japan), and real-time PCR was performed using the KAPA SYBR FAST qPCR Master Mix (Kapa Biosystems, Inc., Wilmington, MA, USA) as previously described [110]. The following primer pairs (synthesized by Eurofins Genomics, Ebersberg, Germany) were used (5′->3′): forward AGGATCAGAGGACAGCACTGCA and reverse AGGTGAAGCCTCCCTGTTGTTC.

#### 4.2.5. Quantification of Nuclear Translocation

The cells co-transfected with GFP-NLS8 and siRNA for IPO8 or negative control siRNA were observed under a confocal microscope (SP8 LIGHTNING Confocal Microscope, Leica Microsystems, Wetzlar, Germany), and representative images were obtained. The fluorescence intensity ratio in the nucleus and the cytoplasm was quantified using ImageJ 1.54 software (Madison, WI, USA, https://imagej.nih.gov/, accessed on 15 January 2025). For each image, the nuclear area was determined in the blue channel of the RGB color system by setting the appropriate threshold area, and the mean fluorescence of the nucleus or cytoplasm was then measured in the green channel. At least 10 images with approximately 40 cells per condition, for three independent experiments, were analyzed. GraphPad Prism 8.0.1 (GraphPad Software Inc., San Diego, CA, USA) was used for statistical analysis. Τhe analysis was parametric and was displayed as mean ± SEM. *p*-values < 0.05 were considered statistically significant.

## 5. Conclusions

In conclusion, the present study highlights the critical RRKLPVGRS recognition motif of importin 8 (IPO8), which localizes to cargo proteins and plays a key role in their transport to the nucleus and their function there. Identification of this importin 8-specific NLS opens the way for future investigations into the development of targeted therapeutic interventions. Indeed, the importin 8-cargo interface could be an appealing target for small molecule- or peptide-based inhibitors in diseases in which importin 8 and its cargo proteins are implicated, including cancer and developmental disorders. This study stresses the necessity of a systematic mapping of NLS sequences among the importin family, as all importins have particular cargo affinities and biological roles. Our findings offer a pragmatic starting point for further endeavors to untangle the whole repertoire of importin–cargo relationships and to investigate their therapeutic potential.

## Data Availability

All data and analysis are available within the manuscript and the Supplementary Materials, or upon request to the corresponding authors.

## Supplementary Materials

The following supporting information can be downloaded at: https://www.mdpi.com/article/10.3390/ijms26062814/s1.
