# Supplementary material for: The Sequence [RRKLPVGRS] Is a Nuclear Localization Signal for Importin 8 Binding (NLS8): A Chemical Biology and Bioinformatics Study
Source: Int J Mol Sci. 2025 Mar 20;26(6):2814. doi: 10.3390/ijms26062814 (PMC11942892; doi:10.3390/ijms26062814)
Supplement: Supplementary file 1 [file ijms-26-02814-s001.zip › ijms-3193449-supplementary.pdf]

The sequence [RRKLPVGRS] is a nuclear localization signal for importin 8 binding (NLS8): a chemical biology and bioinformatics study

Athanasios A. Panagiotopoulos, Konstantina Kalyvianaki, Aikaterini Angelidaki, Dimitris Dellis, Christos A. Panagiotidis, Marilena Kampa and Elias Castanas

Supplementary Material

Contents

Supplementary Tables 2
Table S1 2
Supplementary Figures 3
Figure S1 3
Figure S2 4
Figure S3 5
Figure S4 6
Figure S5 7
Figure S6 8
Figure S7 9
Figure S8 10
Figure S9 11
Figure S10 12
Figure S11 13
Figure S12 14
Figure S13 15
Figure S14 16
Figure S15 17
Figure S16 18
Figure S17 19
Supplementary References 20

Supplementary Tables

Table S1.

Comparison of the identified 3D conformation of proteins that interact with Importin 8. Table presents the protein short name, their PDB code, the total RMSD, and the local RMSD (NLS binding region) between the structures used for *in silico* simulations with the recently reported AlphaFold structures (https://alphafold.ebi.ac.uk/) [1].

Cargo Protein	PDB Code	$RMSD_{total}$	$RMSD_{NLS\ binding\ region}$
AGO1	4KRE	1.124	0.538 (for 181-200 aa)<br>0.117 (for 228-240 aa)<br>0.140 (for 336-350 aa)
AGO2	4F3T	1.229	0.269 (for 166-183 aa)<br>0.424 (for 185-200 aa)<br>0.583 (for 380-396 aa)
AGO3	5VM9	1.373	0.802 (for 186-200 aa)<br>0.339 (for 359-369 aa)<br>0.661 (for 373-387 aa)
AGO4	6OON	0.629	0.158 (for 162-171 aa)<br>0.688 (for 175-185 aa)<br>0.664 (for 362-388 aa)
RPL23A	7OW7	0.780	0.329 (for 10-39 aa)
SMAD1	1KHU (248-465 aa)	0.692	N/A (for 187-199 aa)
SMAD3	5XOC	0.568	0.237 (for 251-273 aa)
SRP19	7NFX	0.881	0.713 (for 45-55 aa)<br>0.508 (for 63-84 aa)<br>0.839 (for 111-126 aa)
TFE3	7F09 (360-430 aa)	1.743	N/A (for 173-214 aa)
WT1	5KL7 (350-440 aa)	1.206	N/A (for 1-9 aa)<br>N/A (for 58-73 aa)<br>0.231 (for 365-378 aa)
ZFP2	N/A	N/A	N/A (for 322-393 aa)<br>N/A (for 418-427 aa)
ZNF264	N/A	N/A	N/A (for 169-185 aa)<br>N/A (for 201-233 aa)<br>N/A (for 236-250 aa)<br>N/A (for 327-338 aa)
ZNF774	N/A	N/A	N/A (for 261-271 aa)<br>N/A (for 280-291 aa)<br>N/A (for 373-380 aa)

Supplementary Figures

Figure S1

Interaction of all the proteins presented in Table 1 and Supplementary Table 1 with Importin 8. Interactions were obtained with the Hex 8.0.0 program, and drawn with Chimera.

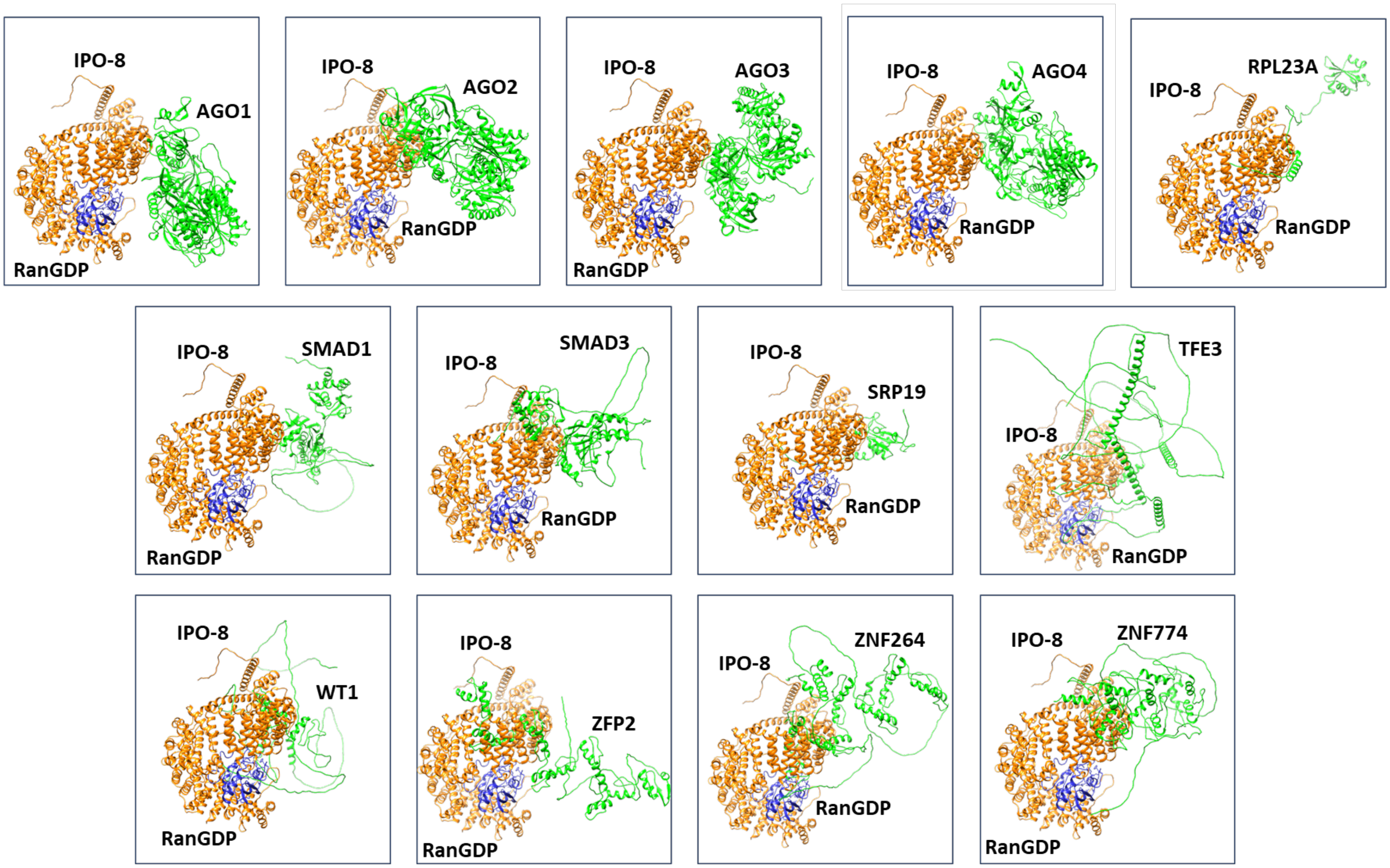

Figure S2

Leave-one-out of AGO1 protein used for IMPO-8-NLS. See main text and Ref [2] for details.

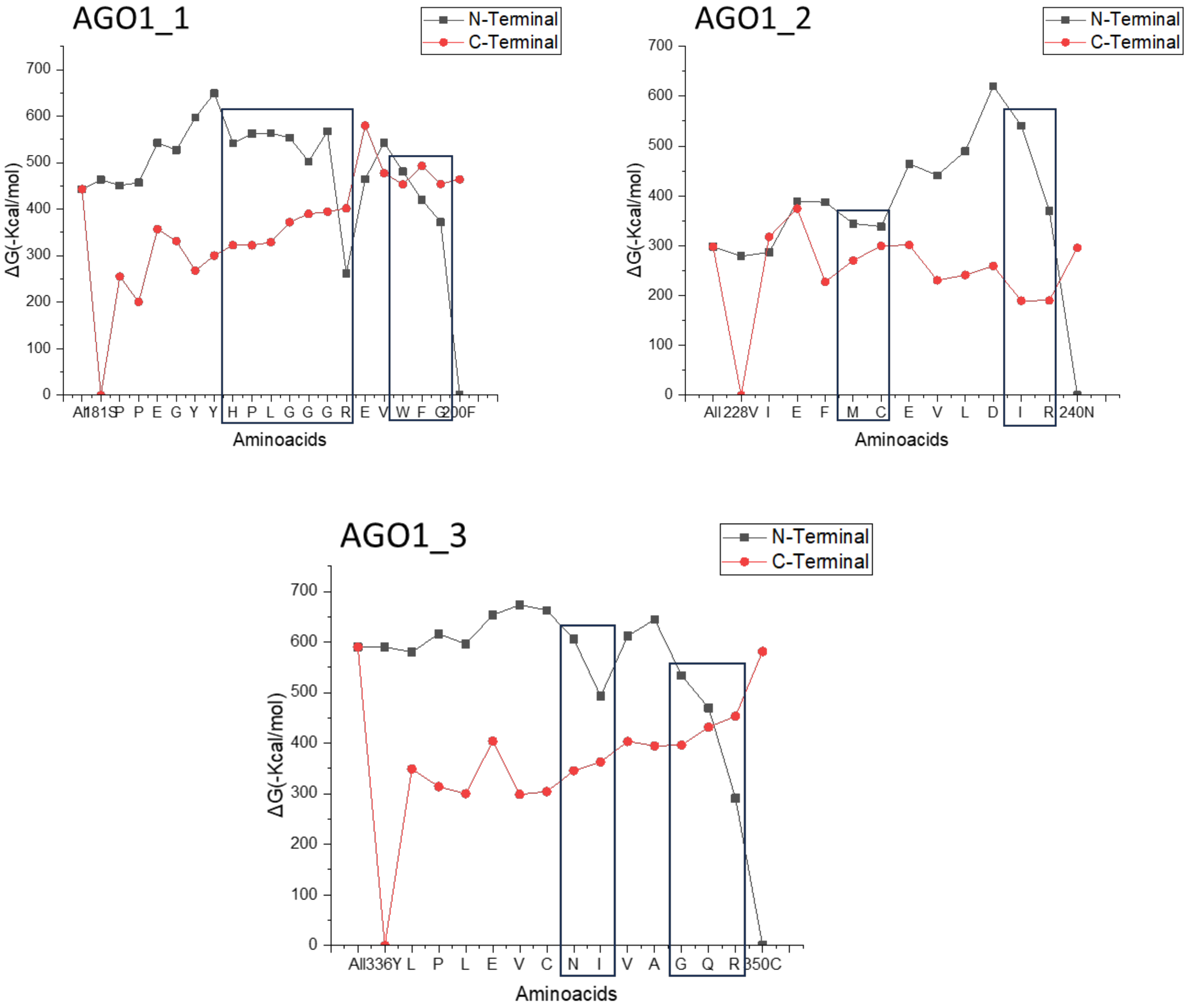

Figure S3

Leave-one-out of AGO2 protein used for IMPO-8-NLS. See main text and Ref [2] for details.

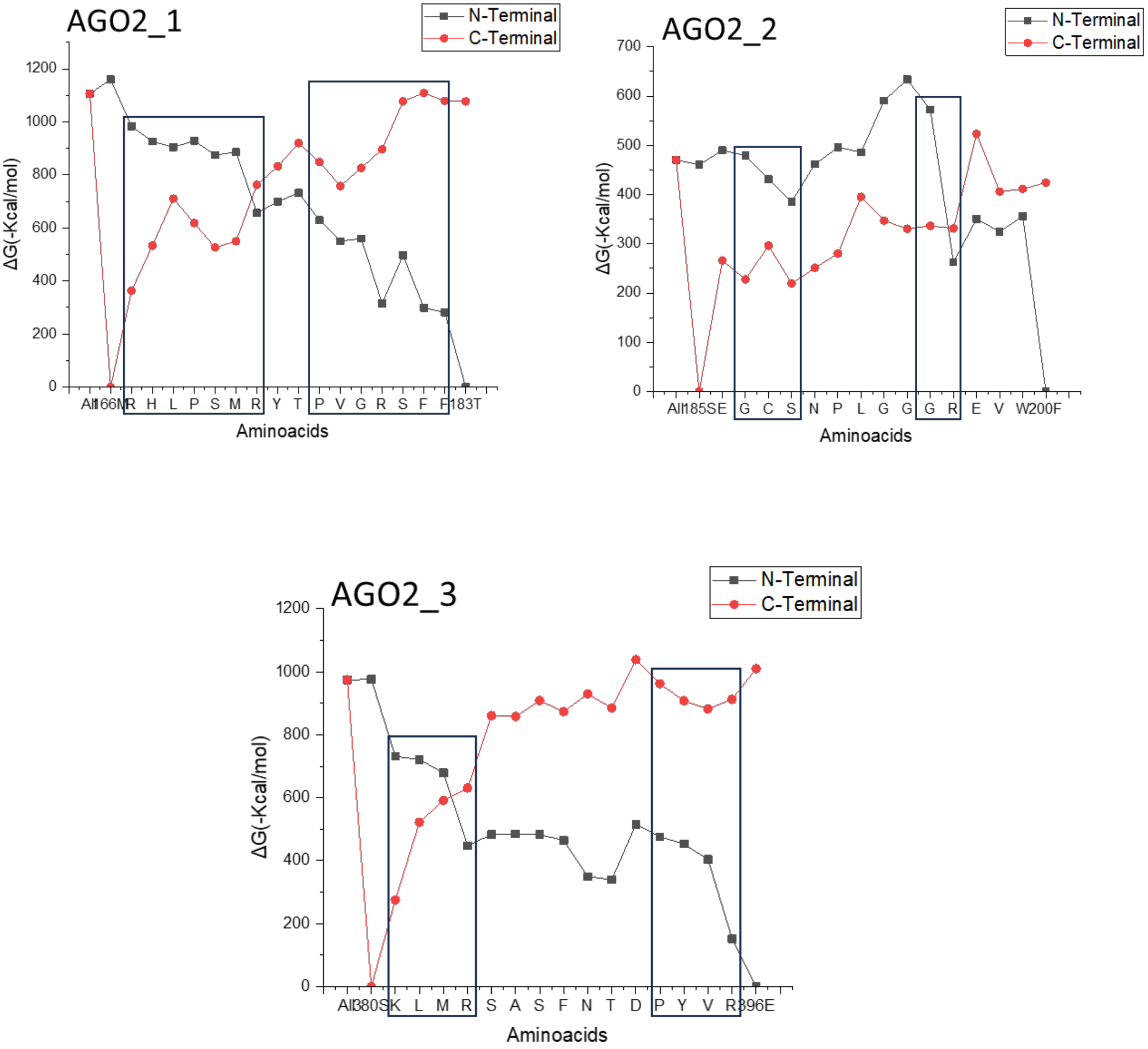

Figure S4

Leave-one-out of AGO3 protein used for IMPO-8-NLS. See main text and Ref [2] for details.

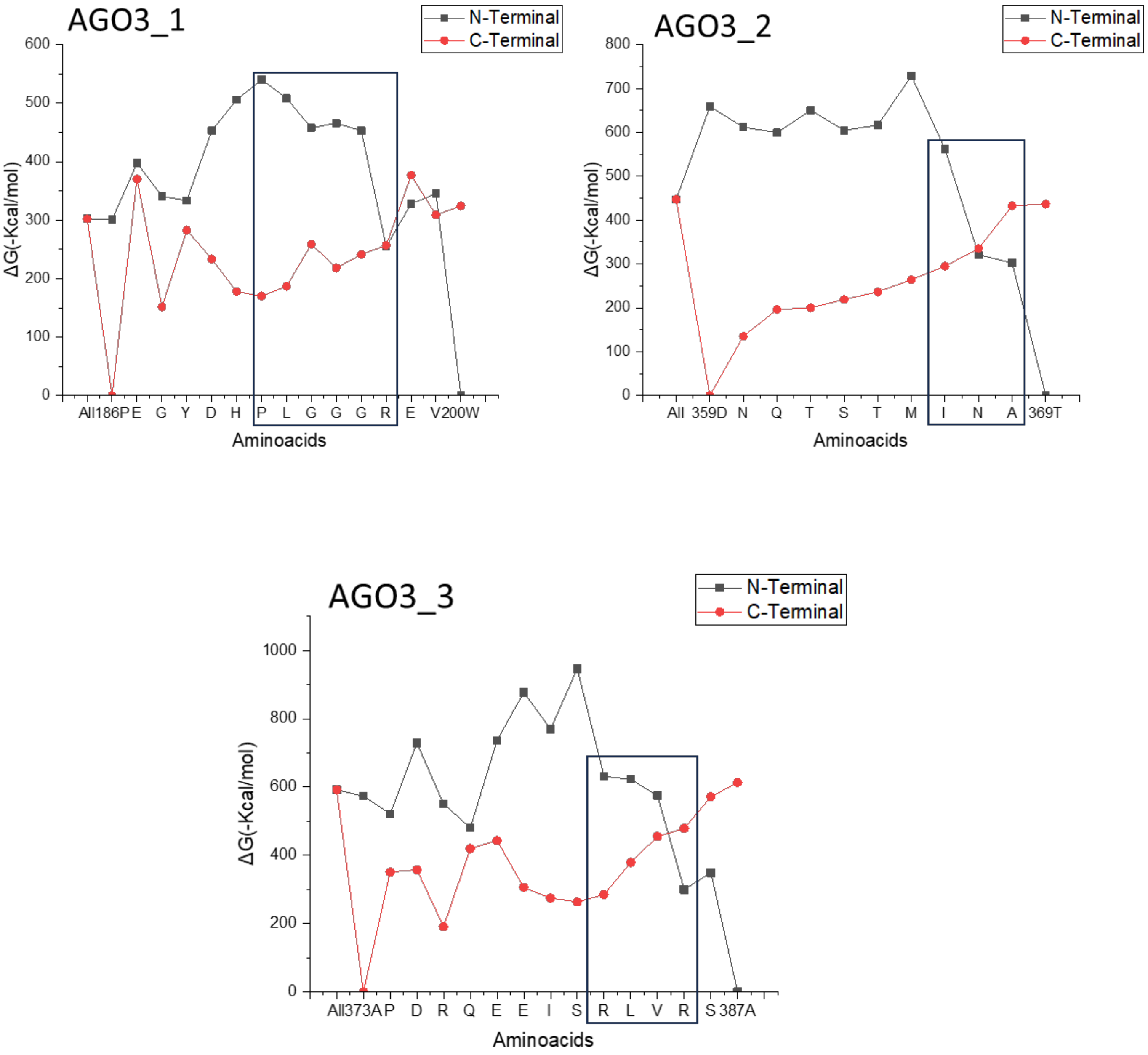

Figure S5

Leave-one-out of AGO4 protein used for IMPO-8-NLS. See main text and Ref [2] for details.

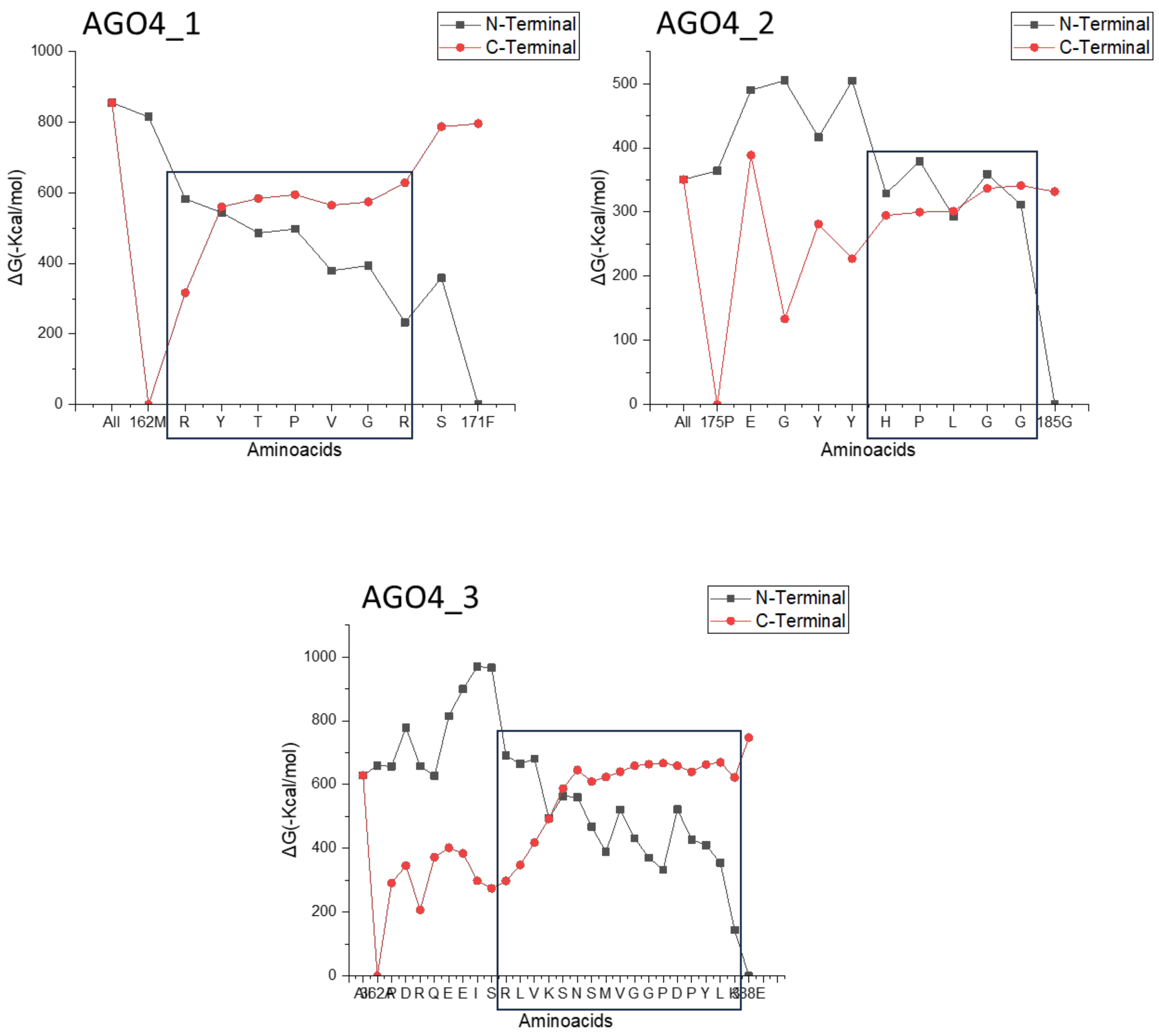

Figure S6

Leave-one-out of RPL23A protein used for IMPO-8-NLS. See main text and Ref [2] for details.

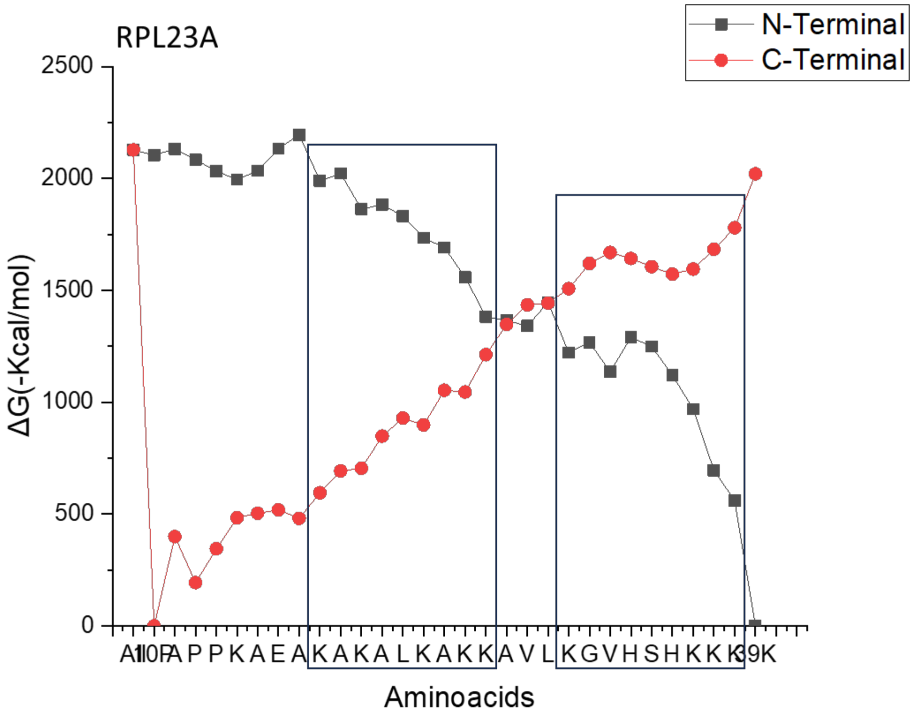

Figure S7

Leave-one-out of SMAD1 protein used for IMPO-8-NLS. See main text and Ref [2] for details.

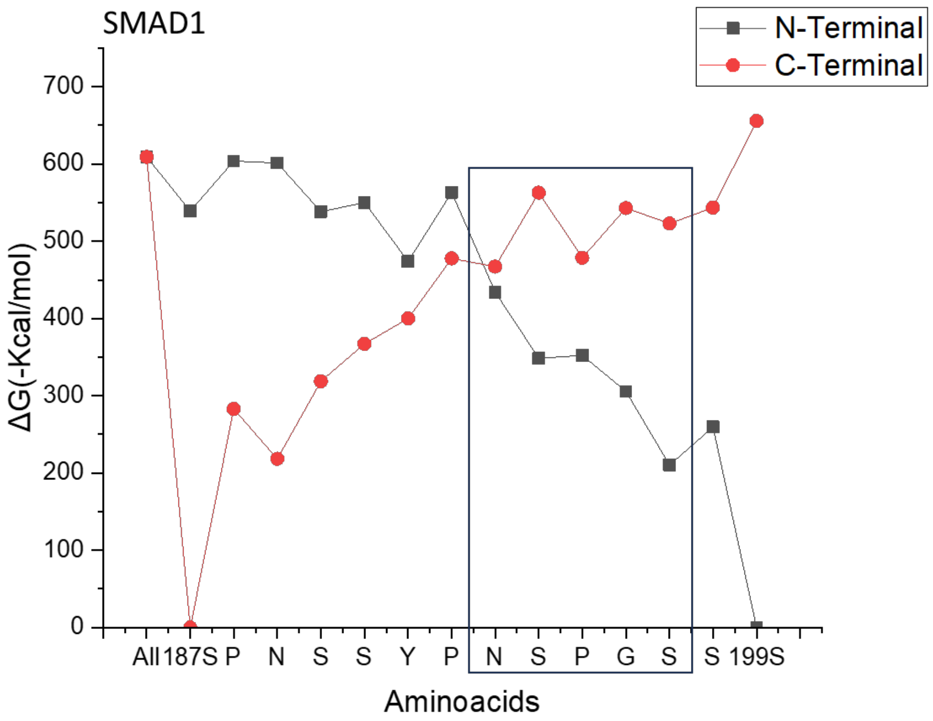

Figure S8

Leave-one-out of SMAD3 protein used for IMPO-8-NLS. See main text and Ref [2] for details.

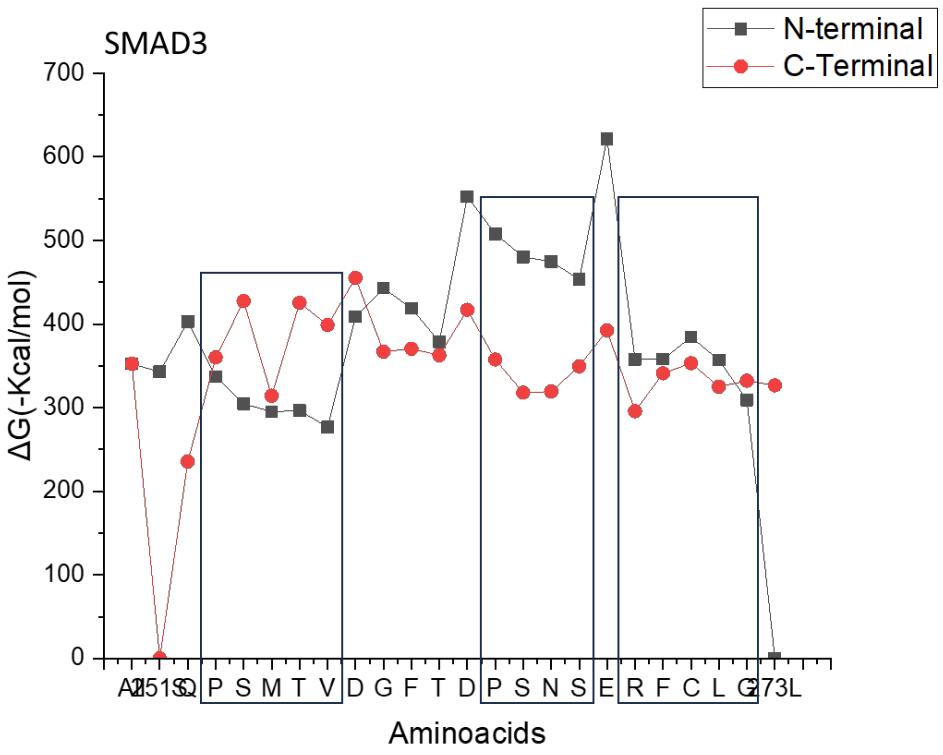

Figure S9

Leave-one-out of SRP19 protein used for IMPO-8-NLS. See main text and Ref [2] for details.

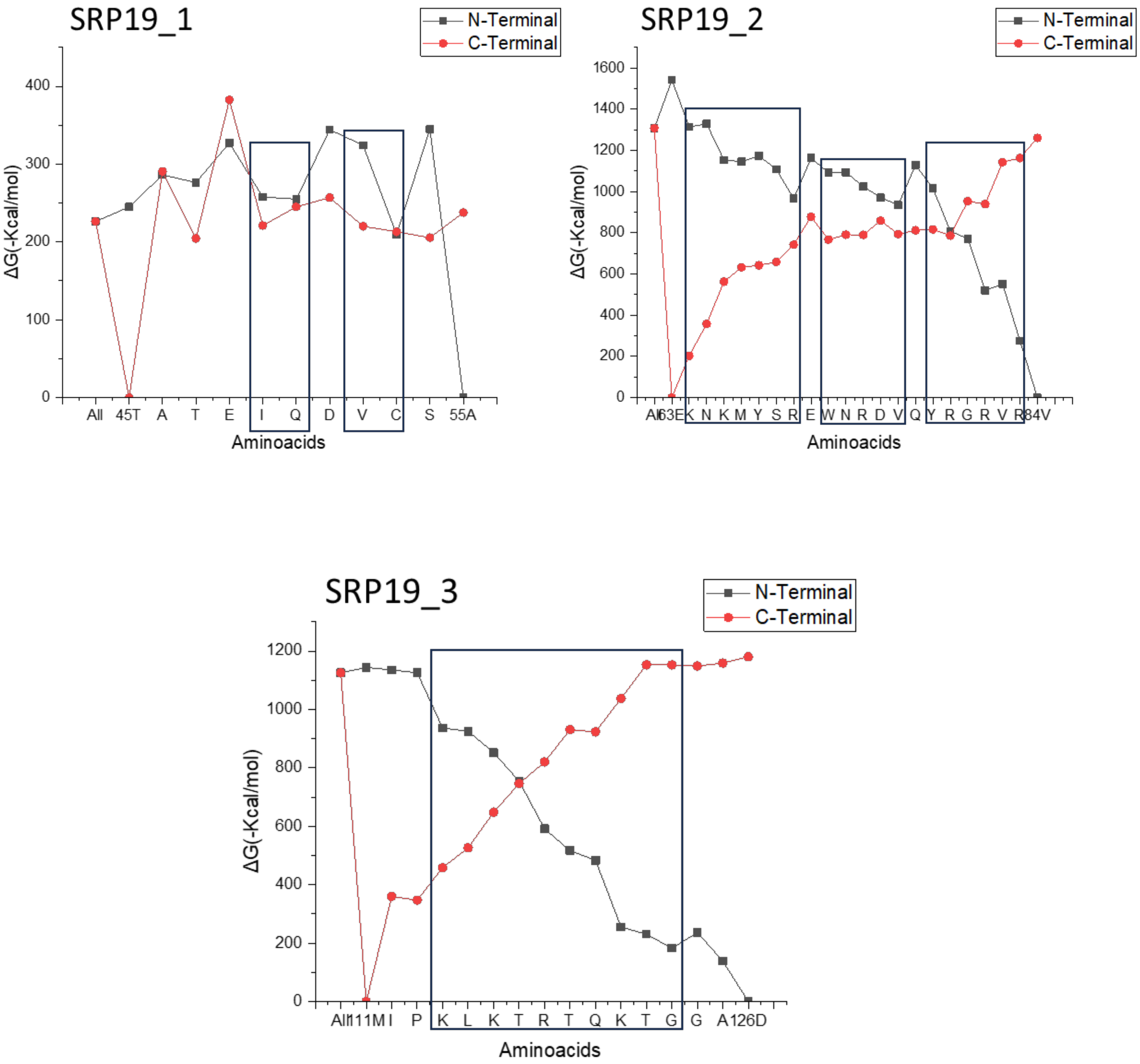

Figure S10

Leave-one-out of TFE3 protein used for IMPO-8-NLS. See main text and Ref [2] for details.

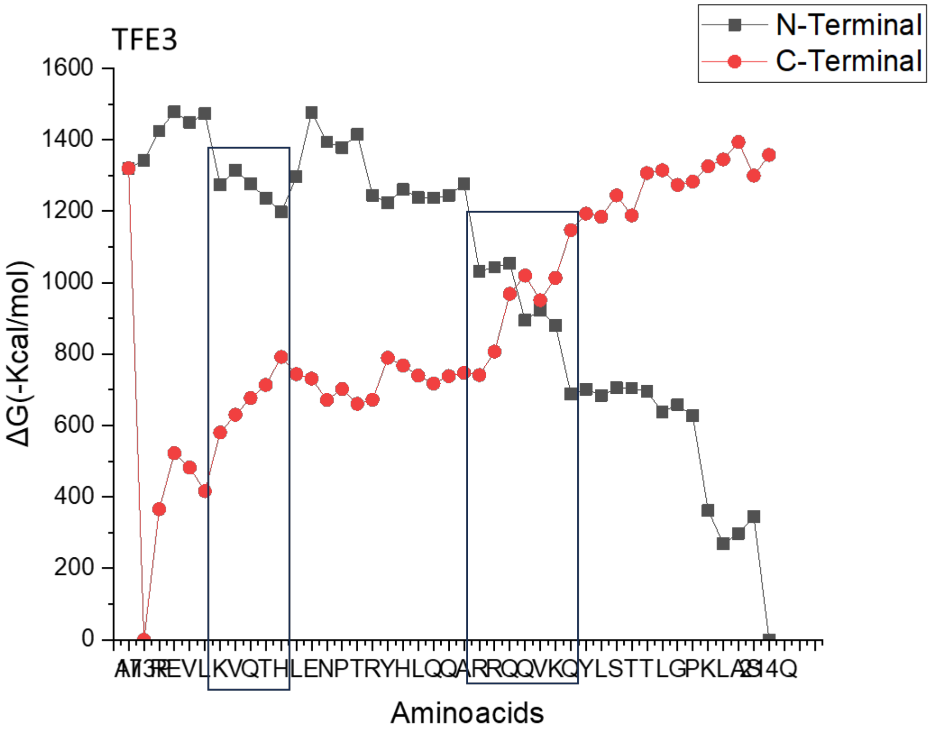

Figure S11

Leave-one-out of WT1 protein used for IMPO-8-NLS. See main text and Ref [2] for details.

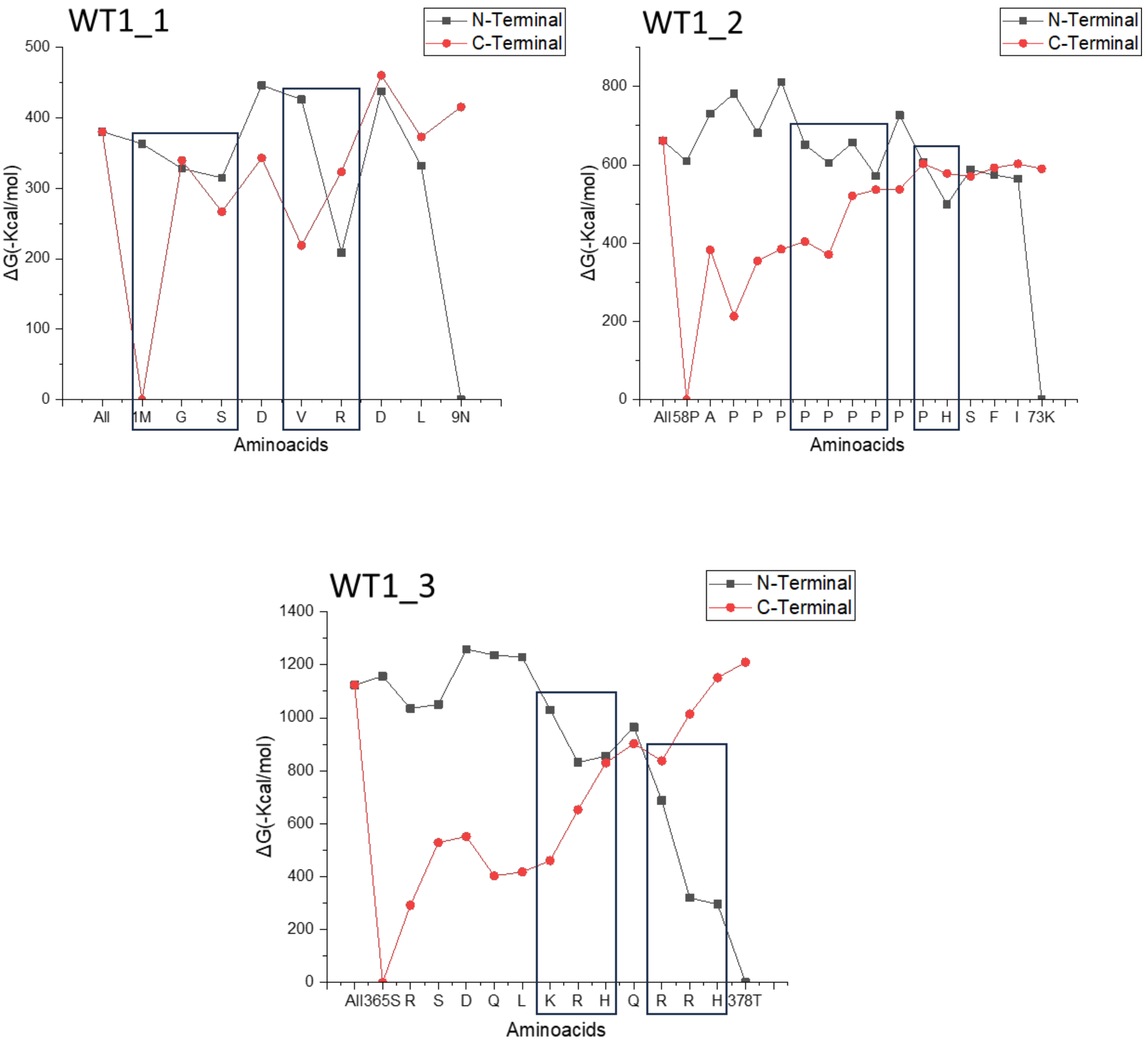

Figure S12

Leave-one-out of ZFP2 protein used for IMPO-8-NLS. See main text and Ref [2] for details.

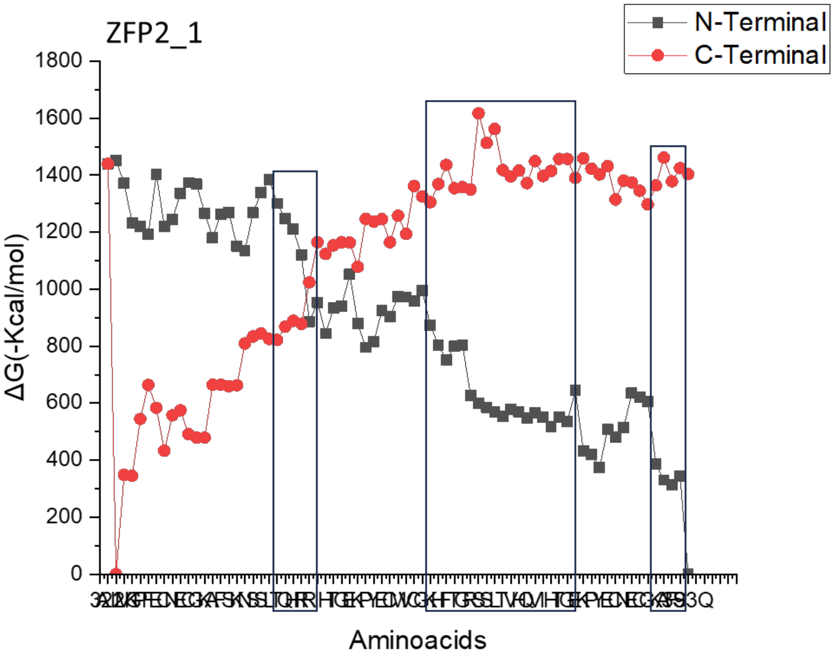

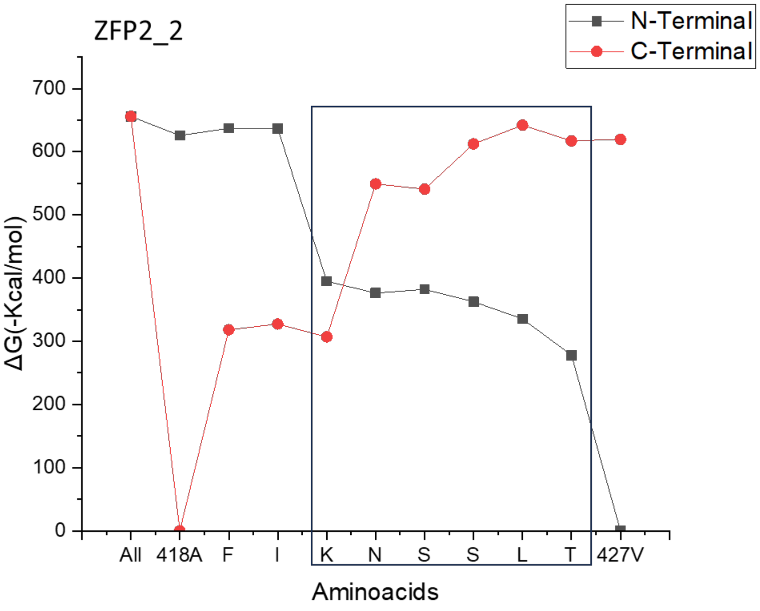

Figure S13

Leave-one-out of ZNF264 protein used for IMPO-8-NLS. See main text and Ref [2] for details.

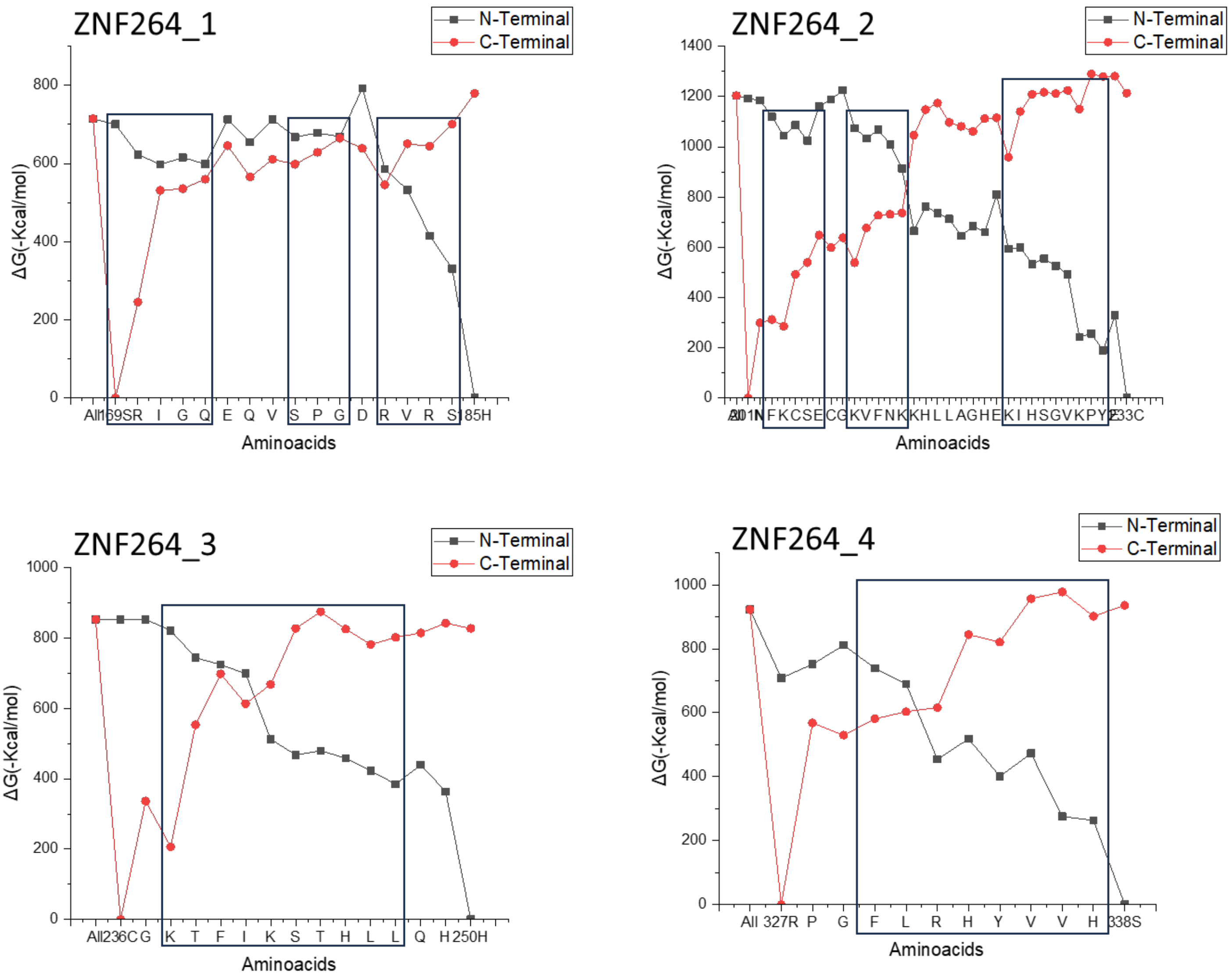

Figure S14

Leave-one-out of ZNF774 protein used for IMPO-8-NLS. See main text and Ref [2] for details.

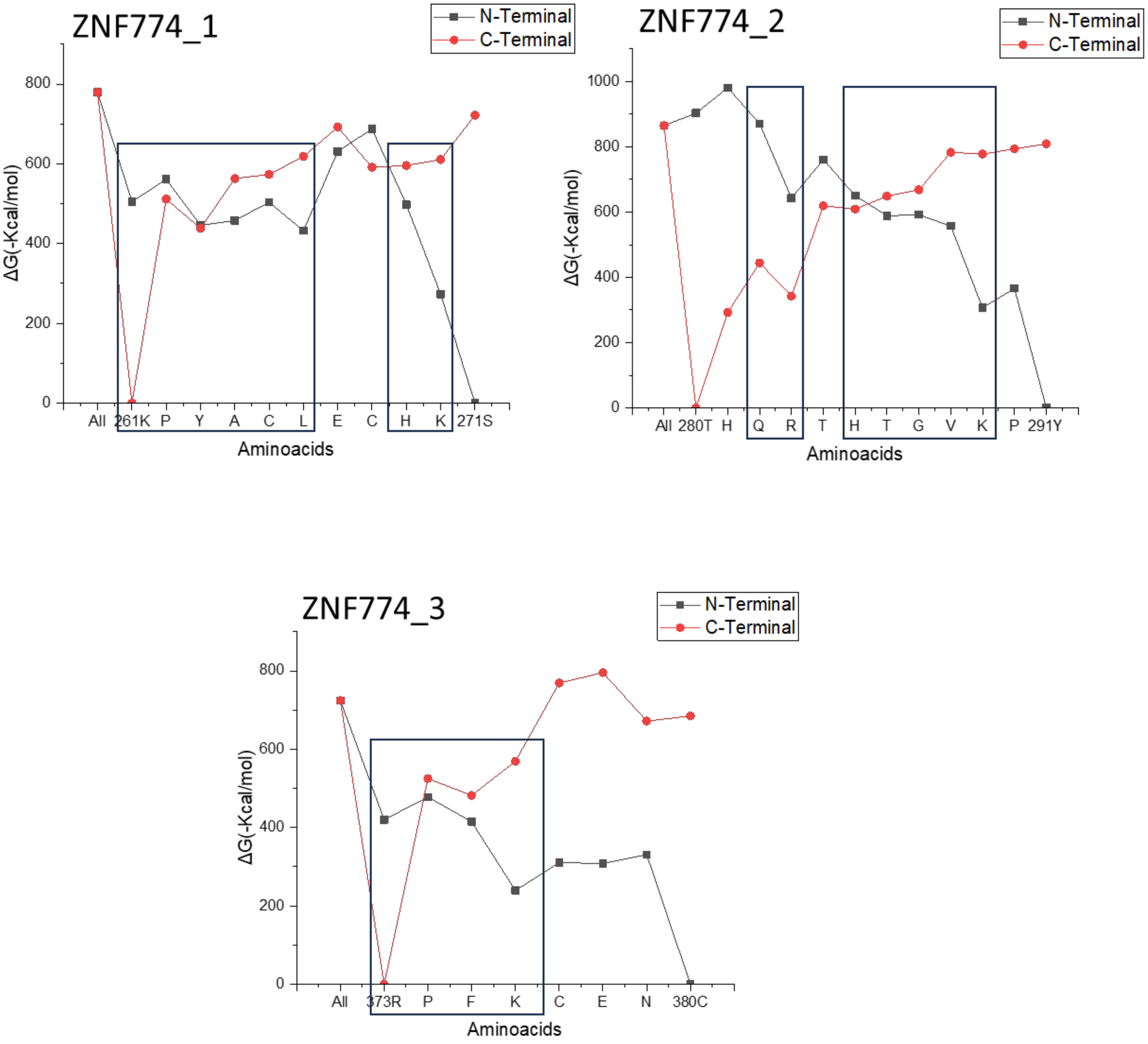

Figure S15

Leave-one-out of NLS8 sequence. See main text and Ref [2] for details.

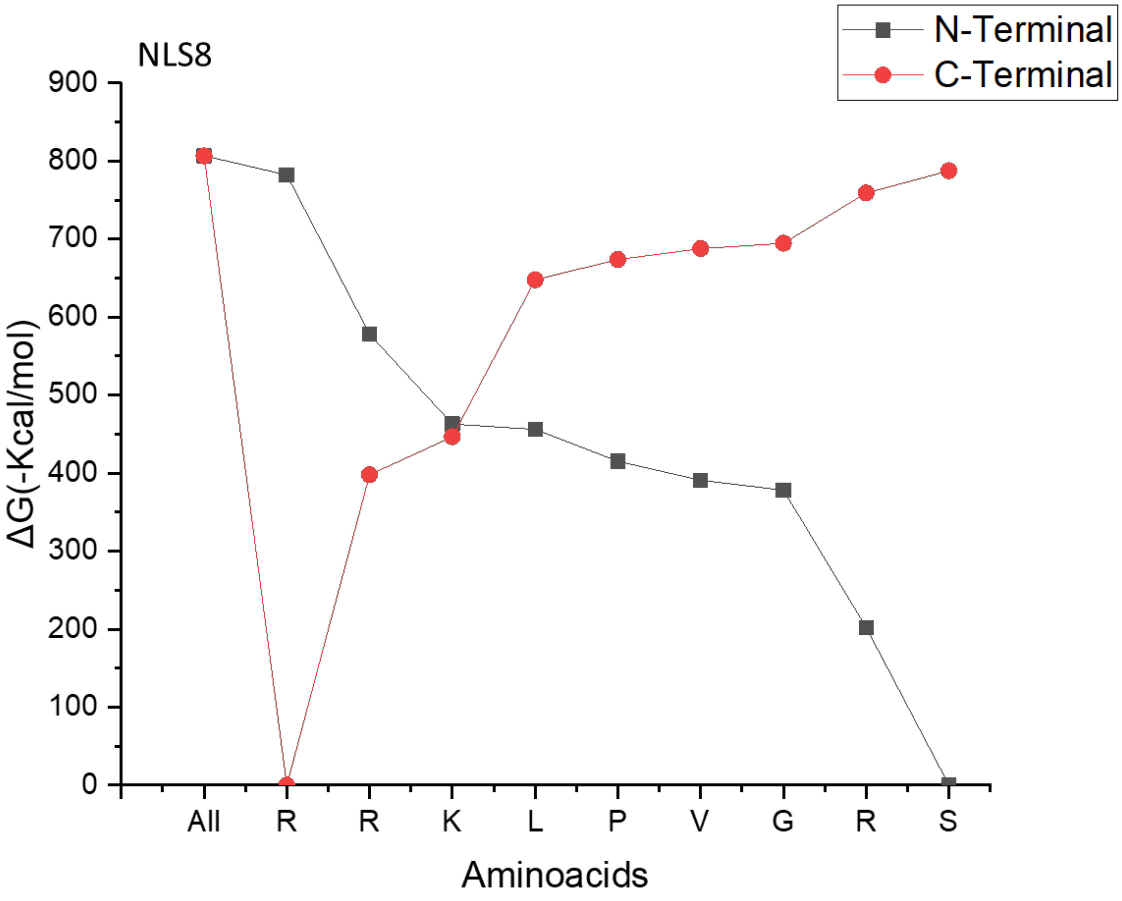

Figure S16

IPO8 expression in various cells. The expression of IPO8 in different cells lines was found using Real Time PCR (A) and (B) "The Human Protein Atlas database".

A.

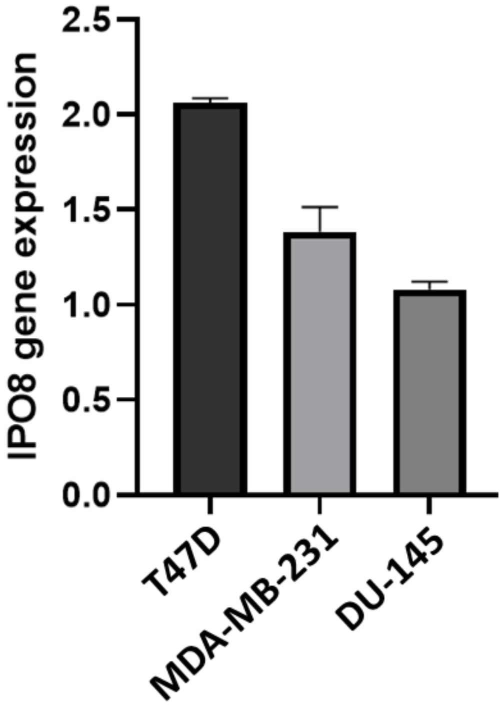

B.

	CELL LINE		
IPO8 RNA EXPRESSION (nTPM)	T47D	MDA-MB-231	DU-145
	45.8	43.1	23.8

Figure S17

Transfection efficiency evaluation. The expression of IPO8 in T47D after transfection with specific siRNAs was assayed by real time qPCR. Results are shown as Mean ± SE of 3 different experiments. Statistical significance vs control *** denotes P<0.001.

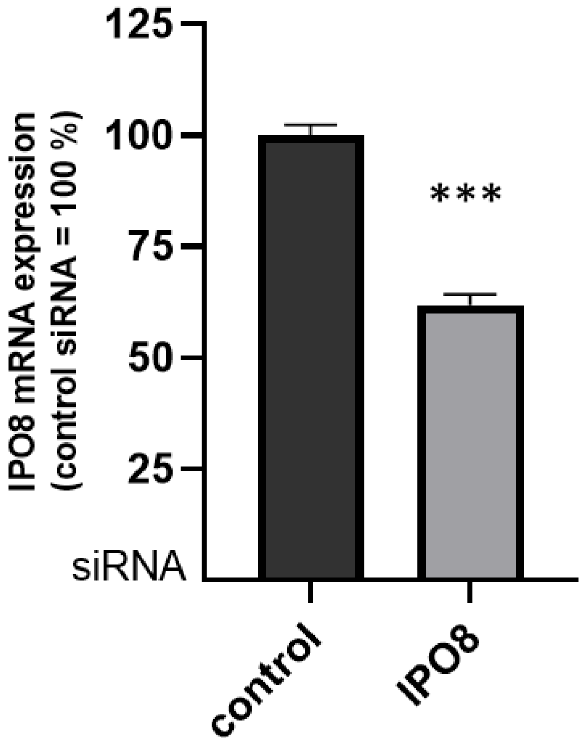

Supplementary References

[1] A.W. Senior, R. Evans, J. Jumper, J. Kirkpatrick, L. Sifre, T. Green, C. Qin, A. Zidek, A.W.R. Nelson, A. Bridgland, H. Penedones, S. Petersen, K. Simonyan, S. Crossan, P. Kohli, D.T. Jones, D. Silver, K. Kavukcuoglu, D. Hassabis, Improved protein structure prediction using potentials from deep learning, Nature, 577 (2020) 706-710.
[2] A.A. Panagiotopoulos, C. Polioudaki, S.G. Ntallis, D. Dellis, G. Notas, C.A. Panagiotidis, P.A. Theodoropoulos, E. Castanas, M. Kampa, The sequence [EKRKI(E/R)(K/L/R/S/T)] is a nuclear localization signal for importin 7 binding (NLS7), Biochim Biophys Acta Gen Subj, 1865 (2021) 129851.
